# Nonstructural 5A Protein of Hepatitis C Virus Interacts with Pyruvate Carboxylase and Modulates Viral Propagation

**DOI:** 10.1371/journal.pone.0068170

**Published:** 2013-07-04

**Authors:** Seung-Ae Yim, Yun-Sook Lim, Jong-Wook Kim, Soon B. Hwang

**Affiliations:** National Research Laboratory of Hepatitis C Virus, Ilsong Institute of Life Science, Hallym University, Anyang, Korea; Pohang University of Science and Technology, Republic of Korea

## Abstract

Hepatitis C virus (HCV) is highly dependent on cellular factors for its own propagation. By employing tandem affinity purification method, we identified pyruvate carboxylase (PC) as a cellular partner for NS5A protein. NS5A interacted with PC through the N-terminal region of NS5A and the biotin carboxylase domain of PC. PC expression was decreased in cells expressing NS5A and HCV-infected cells. Promoter activity of PC was also decreased by NS5A protein. However, FAS expression was increased in cells expressing NS5A and cell culture grown HCV (HCVcc)-infected cells. Silencing of PC promoted fatty acid synthase (FAS) expression level. These data suggest HCV may modulate PC via NS5A protein for its own propagation.

## Introduction

Hepatitis C virus (HCV) is a major causative agent of chronic hepatitis, cirrhosis, and hepatocellular carcinoma (HCC). HCV belongs to the member of the *Hepacivirus* genus within the *Flaviviridae* family. HCV is a positive-sense, single-stranded RNA genome of ∼9.6 kb. The HCV genome encodes a single polyprotein precursor of approximately 3010 amino acids that is cleaved by both cellular signal peptidase and viral protease to generate structural (core, E1, and E2) and nonstructural proteins (p7, NS2, NS3, NS4A, NS4B, NS5A, and NS5B) [1], [2].

The HCV life cycle relies on cellular factors. HCV has been evolved to hijack cellular factors to facilitate replication and virion assembly. Among HCV proteins, NS5A has been implicated in many roles in HCV life cycle, including replication and assembly [3], [4]. In the present study, we identified pyruvate carboxylase (PC) as one of the host factors interacting with NS5A protein by employing tandem affinity purification system. PC catalyzes the ATP-dependent carboxylation of pyruvate to oxaloacetate [5]. PC plays a crucial role in gluconeogenesis and lipogenesis, and its activity is high in the liver, kidney, adipose tissue, and lactating mammary gland [6].

HCV increases triglyceride level in hepatocytes by modulating host metabolism to facilitate its replication and virion release [7], [8]. HCV replication and assembly occur at endoplasmic reticulum and lipid droplets [9], [10]. Lipid droplets, the lipid storage organelles in the cytoplasm, are composed of the neutral lipids surrounded by a monolayer of phospholipids and cholesterol with associated proteins [11]. Hepatic steatosis, the excessive triglyceride accumulation within lipid droplets in the hepatocytes, may be due to metabolic disturbance in HCV infected patients [12]. HCV induces a discrete hepatic steatosis with a prevalence of 34.8% to 81.2%, making this histological finding two to three times more common than liver diseases caused by other etiologic agent [13]. However, pathological mechanisms of HCV-induced liver steatosis are not clearly understood. In the present study, we showed that NS5A interacted with PC through the N-terminal region of NS5A and the biotin carboxylase domain of PC and this interaction was observed in cell culture grown HCV (HCVcc)-infected cells. We showed that PC expression level was decreased, whereas fatty acid synthase (FAS) expression level was increased in cells expressing NS5A protein. Taken together, HCV may modulate lipogenesis by hijacking PC via NS5A protein to facilitate its own propagation.

## Materials and Methods

### Plasmids and DNA Transfection

Myc-tagged wild-type and mutants of NS5A expression plasmids were generated by PCR using the genotype 1b of HCV as a template and subcloned into the pEF6A (Invitrogen, Carlsbad, California) or pNTAP (Stratagene, La Jolla, California) vector. cDNA encoding human PC was amplified from the pOTB7-PC plasmid (21C Frontier Gene Bank, Korea) and subcloned into the pFlag-CMV2 (Sigma-Aldrich, ST. Louis, Missouri) or pEF6-His vector. PC mutants were generated by PCR and subcloned into the pFlag-CMV vector. Stable cells expressing NS5A protein were selected as described previously [14].

### Cell Culture and Virus Infection

All cell lines were grown in Dulbecco’s modified Eagle’s medium (DMEM) supplemented with 10% fetal calf serum and 1% penicillin/streptomycin. HCV subgenomic replicon and IFN-α cured cells were grown as we reported previously [15]. The infectious HCVs generated as described previously [16], [17] were used to infect Huh7.5 cells.

### Tandem Affinity Purification (TAP)

Huh7.5 cell transfected with either pNTAP empty vector or pNTAP-NS5A vector were harvested at 48 h after electroporation. Cells were lysed and then TAP-tagged protein and its associated proteins were purified according to the manufacturer’s protocol (Stratagene). Proteins copurified with TAP-NS5A were separated on an 8% SDS-PAGE and visualized by silver staining. The interested protein bands were excised and analyzed by liquid chromatography-tandem mass spectrometry (LC/MS/MS). The generated peak list files were used to query either the MSDB data base or NCBI using the MASCOT program.

### Quantitative Real-time PCR Analysis

Both intracellular and extracellular RNAs were isolated from HCVcc-infected cells, cell culture media, or replicon cells using either TRIzol® or TRIzol® LS reagent (Invitrogen) and were reverse transcribed using iScriptTM cDNA synthsis kit (Bio-Rad Laboratories, Hercules, CA). Quantitative real-time PCR (qRT-PCR) experiments were performed using an iQ SYBR® Green Supermix (Bio-Rad Labotories) and an iQ5 multicolor real-time PCR detection system (Bio-Rad Laboratories). To estimate RNA levels of HCV and PC, cDNA was amplified with genotype 2a specific primers (forward, 5′-TTA GTA TGA GTG TCG TAC AGC CTC CAG-3′; reverse, 5′-GGC ATA GAG TGG GTT TAT CCA AGA AAG G-3′), genotype 1b specific primers (forward, 5′-ATC ACT CCC CTG TGA GGA ACT ACT G-3′; reverse, 5′-CTG GAG GCT GCA CGA CAC TC-3′), GAPDH primers (forward, 5′-CGC TCT CTG CTC CTC CTG TTC-3′; reverse, 5′-CGC CCA ATA CGA CCA AAT CCG-3′), and PC primers (forward, 5′-GCA CTA CTT CAT CGA GGT CAA CT-3′; reverse, 5′-CGT GGA TCT GAG CAT GGA C-3′). The HCV 5′NTR was cloned into T&A cloning vector (RBC Bioscience Corp., New Taipei, Taiwan) and was used as a standard for qRT-PCR. The standards were run in parallel with the samples and the RNA copy numbers were calculated based on the standard curve.

### Intracellular and Extracellular Infectivity Assays

Huh7.5 cells were treated with the indicated siRNA and were infected with HCV at 2 days after transfection. The media were collected at 2 days postinfection to determine the infectivity of extracellular HCV. The remaining cells were suspended in 1 ml PBS and were lysed by four rounds of freeze-thaw cycles. The cell lysates were clarified by centrifugation at 3,400 rpm for 5 min. The clear supernatant was collected and was used to determine the infectivity of intracellular HCV. Naïve Huh7.5 cells were infected with either intracellular HCV or extracellular HCV. HCV RNA levels were determined by qRT-PCR at 2 days postinfection.

### RNA Interference

PC SMARTpool siRNA and universal negative control siRNA (ON-TARGETplusTM) were purchased from Dharmacon (Lafayette, CO). siRNA targeting 5′NTR of Jc1 (5′-CCU CAA AGA AAA ACC AAA CUU-3′) was used as a positive control [18]. siRNA transfection was performed using a Lipofectamine RNAiMax reagent (Invitrogen, Carlsbad, CA) according to the manufacturer’s instructions.

### Confocal Microscopy

Huh7.5 cells infected with HCVcc were fixed in 4% paraformaldehyde containing 0.1% Triton X-100. Cells were incubated with an anti-Flag antibody (Sigma), an anti-PCB antibody (Santa Cruz Biotechnology Inc., Santa Cruz, California), an anti-NS5A antibody (kindly provided by Dr. Ahn, Korea University, Korea), or an anti-VDAC antibody (Santa Cruz) for 2 h. After three washes with PBS, cells were further incubated with the appropriate secondary antibody and analyzed using the Zeiss LSM 700 laser confocal microscopy system (Carl Zeiss, Inc., Thornwood, NY).

### MTT Assay

The 3-(4,5-cimethylthiazol-2-yl)-2,5-diphenyl tetrazolium bromide (MTT, Sigma) assay was performed to determine the host cell viability in siRNA transfected cells according to the manufacturer’s instructions.

### Immunoblot Analysis

Immunoblot assay was performed as we described previously [16]. Equal amounts of proteins were subjected to SDS-PAGE and were electrotransferred to a nitrocellulose membrane. The membrane was blocked in Tris-buffered saline-Tween (20 mM Tris-HCl [pH 7.6], 150 mM NaCl, and 0.25% Tween 20) containing 5% nonfat dry milk for 1 h and then incubated overnight at 4°C with the indicated antibodies in Tris-buffered saline-Tween containing 1% nonfat dry milk. Following three washes in Tris-buffered saline-Tween, the membrane was incubated with either horseradish peroxidase-conjugated goat anti-rabbit antibody or goat anti-mouse antibody (Jackson ImmunoResearch Laboratories, Inc., West Grove, PA) in Tris-buffered saline-Tween containing 1% nonfat dry milk for 1 h at room temperature. Proteins were detected using an ECL kit (Amersham Biosciences, Piscataway, NJ).

### Cloning of PC Promoter Constructs and Reporter Assays

Human genomic DNA was isolated from Huh7.5 cells and PC promoters were amplified using PCR with the specific primers. The primers of PC promoter 1 (from −2000 to +18) are 5′-AGA TCT AGA AAC TGC ATG GTT TGT G-3′ and 5′-TAA AAG CTT AGG CCA CAC TGT TCC-3′. The primers of PC promoter 2 (from −1938 to +20) are 5′-ATA CGC GTT CAG ATC TCT CCA GGC TCA AT-3′ and 5′-AAA TAC TCG AGG CTG CTG CCT CCA CTG A-3′. The amplified PCR products were cloned into pGL3 vector (Promega Corp., Madison, WI) to generate luciferase reporter construct of either pGL3-PC-P1 (PC promoter 1) or pGL3-PC-P2 (PC promoter 2). Luciferase and β-galactosidase assays were performed as described previously [14].

### Isolation of Mitochondria

Mitochondrial and cytoplasmic cell fractions were isolated by Qproteome mitochondria isolation kit (QIAGEN, Crawley, UK). Briefly, Huh7.5 cells infected with HCVcc were cultured for 2 days. Cells (2×10^7^) were trypsinized, centrifuged at 500 × g for 10 min at 4°C, and washed with buffer containing 0.9% sodium chloride. Cells were resuspended in ice-cold lysis buffer for 10 min at 4°C and centrifuged at 1000 × g for 10 min at 4°C. The supernatant was further centrifuged at 13,000 × g for 30 min to obtain soluble cytosolic proteins. The cell pellet was resuspended in ice-cold buffer and completely disrupted by using a blunt-ended needle and a syringe. The lysates were centrifuged at 1,000 × g for 10 min and then supernatant was further centrifuged at 6,000 × g for 10 min. The supernatant constitutes the microsomal fractions. The pellet was resuspended in mitochondrial purification buffer and carefully loaded on the top layer of purification buffer and disruption buffer. High-purity mitochondria were obtained by centrifugation at 14,000 × g for 15 min at 4°C. Subcellular fractions were verified by immunoblot analysis using the corresponding antibodies.

### Statistical Analysis

The data are presented as mean ± SD. The student *t* test was performed for statistical analysis. P<0.05 was considered statistically significant. Error bars represent SDs of three independent experiments.

## Results

### Identification of PC as a Cellular Factor Interacting with HCV NS5A Protein

**Figure 1:**
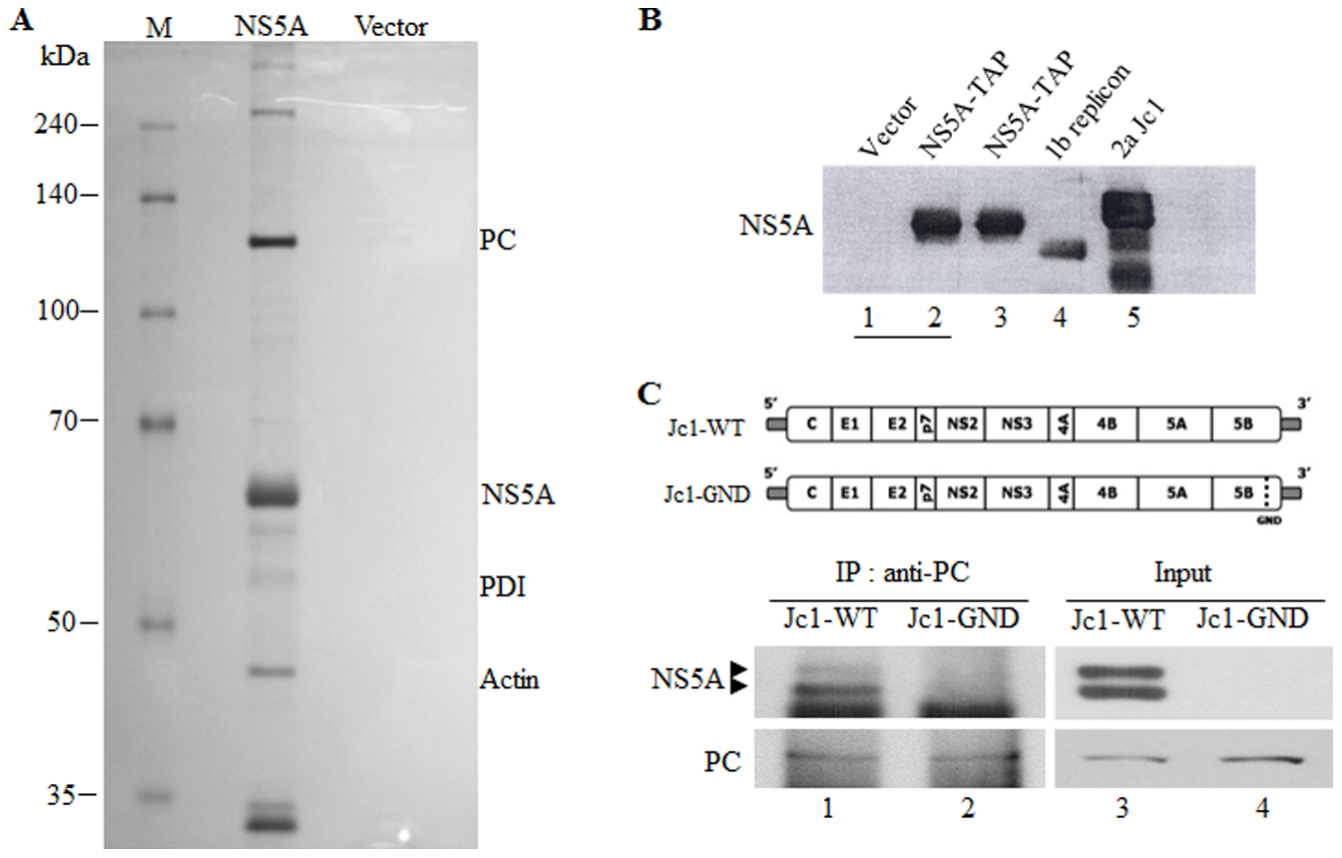
Identification of cellular factors interacting with HCV NS5A. (A) Purification of cellular proteins interacting with NS5A. Huh7.5 cells were electroporated with either pNTAP empty vector or pNTAP-NS5A plasmid. At 3 days after electroporation, cells were harvested and then TAP-tagged NS5A and its associated protein complexes were purified as described in Materials and Methods. The interested protein bands were excised from the gel and analyzed by LC-MS/MS. (B) Verification of NS5A protein used in tandem affinity purification. The protein complexes purified from cells transfected with either pNTAP empty vector (lane 1) or pNTAP-NS5A expression plasmid (lane 2) were immunoblotted with NS5A antibody. Total cell lysates harvested from TAP-tagged NS5A transfected cells (lane 3), HCV 1b replicon cells (lane 4), and HCV Jc1-infected cells (lane 5) were immunoblotted with NS5A antibody. (C) Coimmunoprecipitation of PC and NS5A in HCV-infected cells. The diagram represents both wild-type and replication defective mutant of Jc1 (upper panel). Huh7.5 cells were transfected with 10 µg of either wild type or mutant Jc1 RNA. Cell lysates harvested at 4 days after transfection were immunoprecipitated with PC antibody and then bound protein was detected by immunoblot analysis using NS5A antibody (lower panel).

NS5A is a multifunctional protein involved in HCV propagation. To identify cellular proteins interacting with HCV NS5A protein, Huh7.5 cells were electroporated with either pNTAP empty vector or pNTAP-NS5A vector and were harvested at 3 days after electroporation. NS5A interacting proteins were purified by tandem affinity purification method, resolved on an 8% SDS-PAGE, and visualized by the silver staining. As shown in Fig. 1A, several cellular proteins with various molecular masses were co-purified with NS5A protein. Each band was sliced from the gel and analyzed by LC-MS/MS. Approximately 130 kDa protein was identified as the PC, ∼55 kDa protein as the protein disulfide isomerase (PDI), and ∼45 kDa protein as the actin. Purified NS5A protein was also verified by immunoblot analysis using rabbit anti-NS5A antibody (Fig. 1B). Because PC was considered as a crucial enzyme involved in HCV-mediated lipid accumulation, we selected PC for further study. To verify protein interaction between NS5A and PC in HCVcc-infected cells, Huh7.5 cells transfected with either wild-type or GND mutant of Jc1 RNA were immunoprecipitated with PC antibody. Indeed, we showed that both hypophosphorylated (p56) and hyperphosphorylated (p58) forms of HCV NS5A were coimmunoprecipitated with PC (Fig. 1C, lane 1).

### NS5A Interacts with PC Through the Biotin Carboxylase Domain of PC and the Domain I of NS5A

**Figure 2:**
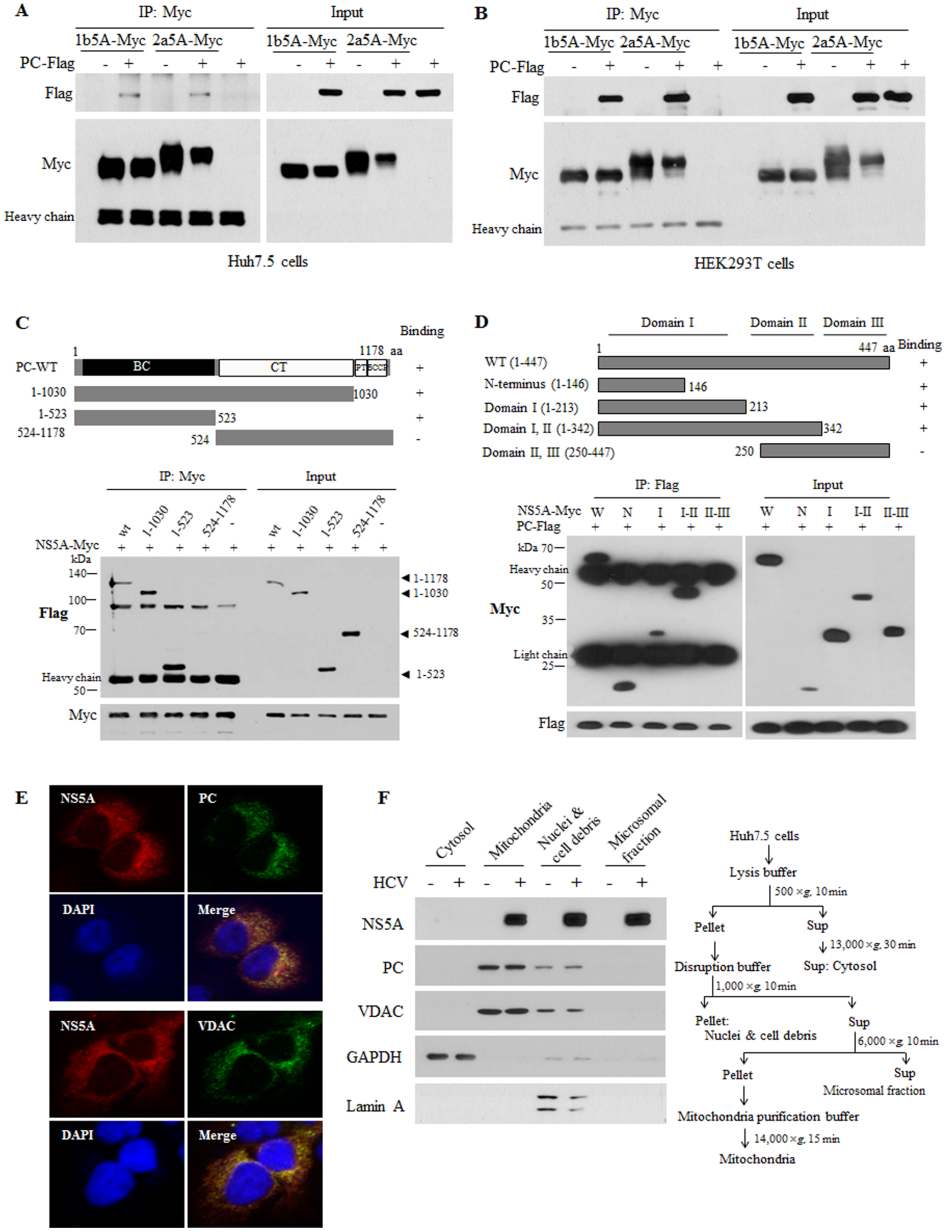
PC interacts and colocalizes with the HCV NS5A protein. PC interacts with NS5A derived from both genotype 1b and 2a. Either Huh7.5 cells (A) or HEK293T cells (B) were cotransfected with Myc-tagged NS5A of genotype 1b or 2a in the absence or presence of Flag-tagged PC. Cell lysates harvested at 24 h after transfection were immunoprecipitated with anti-Myc antibody and then bound proteins were immunoblotted with anti-Flag antibody. Protein expressions of NS5A and PC were verified using the same cell lysates by immunoblotting with anti-Myc antibody and anti-Flag antibody, respectively. (C) The biotin carboxylase domain of PC is the binding site for NS5A. Schematic diagram of both wild type and mutants of PC (upper panel). HEK293T cells were cotransfected with Myc-tagged NS5A and Flag-tagged mutant constructs of PC. Cells were harvested at 24 h after transfection and were immunoprecipitated with anti-Myc antibody. The coprecipitated proteins were immunoblotted with anti-Flag antibody (lower panel). BC, biotin carboxylase; CT, carboxyltransferase; PT, PC tetramerization; BCCP, biotin-carboxyl carrier protein. (D) PC interacts with the domain I of NS5A. Schematic diagram shows both wild type and mutants of NS5A (upper panel). HEK293T cells were cotransfected with Flag-tagged PC and Myc-tagged constructs of NS5A. Cell lysates harvested at 24 h after transfection were immunoprecipitated with anti-Flag antibody and then bound proteins were immunoblotted with anti-Myc antibody. Protein expressions of both NS5A and PC were confirmed using the same lysates by immunoblotting with anti-Myc and anti-Flag antibodies, respectively. (E) PC colocalizes with NS5A. Huh7.5 cells were infected with HCV Jc1 for 4 h. At two days postinfection, cells were fixed and then incubated with anti-NS5A, anti-PC antibody, and anti-VDAC antibody, respectively. Cells were further incubated with the appropriated secondary antibodies and then counterstained with 4′,6-diamidino-2-phenylindole (DAPI) to visualize nuclei. (F) NS5A localizes to mitochondria. Huh7.5 cells were infected with Jc1 for 4 h and were cultured for 2 days. The cells were fractionated into cytosol, mitochondria, nuclei & cell debris, and microsomal fraction. The purities of the each subcellular fraction were confirmed by using GAPDH, VDAC, and Lamin A antibody, respectively.

We confirmed that PC interacted with NS5A derived from both genotype 1b and 2a in both Huh7.5 (Fig. 2A) and HEK293T cells (Fig. 2B). To determine which domain of PC was responsible for binding with NS5A, several deletion mutants of PC were constructed based on functional domains (Fig. 2C, upper panel). HEK293T cells were cotransfected with Myc-tagged NS5A (genotype 1b) and either wild type or mutants of PC, and then interaction was determined by a coimmunoprecipitation assay. As shown in Fig. 2C, NS5A interacted with the biotin carboxylase domain of PC (lower panel). We next determined the region in NS5A responsible for PC binding using various truncated mutants of NS5A (Fig. 2D, upper panel). Fig. 2D showed that PC interacted with NS5A through the N-terminal region (1–146) of NS5A (lower panel).

### A Fraction of NS5A Colocalizes with PC in the Mitochondria of Hepatocytes

Protein interplay data suggest that PC may colocalize with NS5A. To investigate this possibility, Huh7.5 cells infected with Jc1 were examined for the subcellular localization of PC and NS5A by confocal microscopy. Fig. 2E showed that both NS5A and PC were localized in the cytoplasm, and dual staining revealed that both proteins were colocalized in the cytoplasm as yellow fluorescence in HCV-infected cells. We also demonstrated that NS5A colocalized with mitochondria by staining cells with mitochondrial-specific marker, VDAC (Fig. 2E). We further verified that NS5A and PC were comigrated in the mitochondrial fraction in Jc1-infected cells (Fig. 2F). In fact, NS5A is a membrane-anchored phosphoprotein that possesses multiple and diverse functions [19]. It was noteworthy that NS5A was localized not only in the ER fraction but also in the mitochondrial fraction in which PC was mainly presented (Fig. 2F). These data collectively provide the evidence that the interaction between NS5A and PC was occurred in HCVcc-infected cells.

### Silencing of PC Impairs Virion Packaging and Release

**Figure 3:**
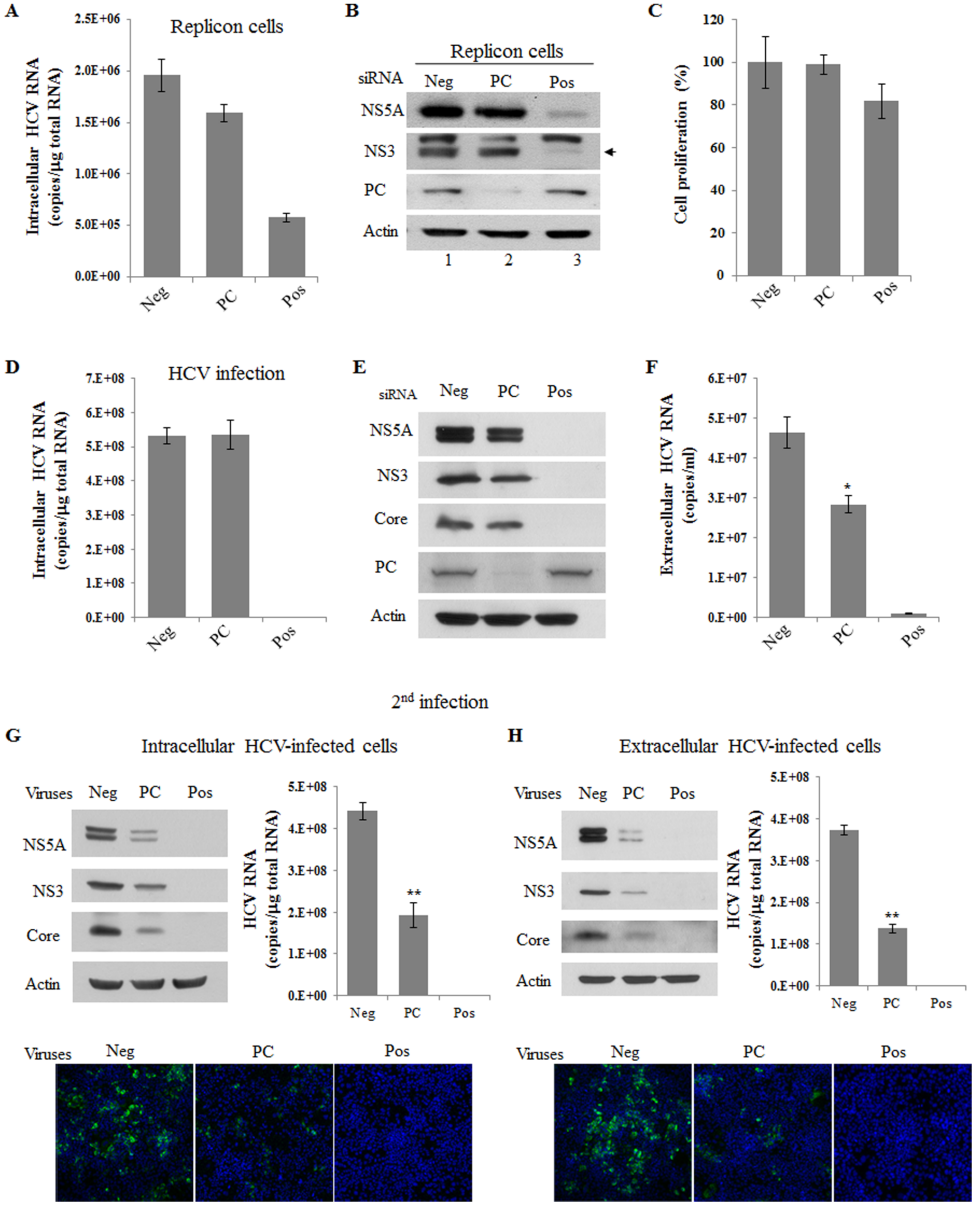
Silencing of PC impairs production of infectious HCV. (A) Knockdown of PC had no effect on viral protein levels in HCV replicon cells. Huh7 cells harboring HCV replicon were transfected with 10 nM of negative (Neg), positive (Pos), or the indicated siRNA duplexes for 3 days. Total RNAs were extracted and intracellular HCV RNA was analyzed by qRT-PCR. Negative, irrelevant siRNA pool; positive, HCV-specific siRNAs. (B) Total replicon cell lysates harvested at 3 days after siRNA transfection were immunoblotted with the indicated antibodies. (C) Huh7.5 cells were transfected with 10 nM of the indicated siRNA. At 2 days after siRNA transfection, cells were infected with Jc1 for 4 h. At 48 h postinfection, cell proliferation was assessed by the MTT assay. Huh7.5 cells were treated as described in (C). At 48 h postinfection, both intracellular HCV RNA (D) and extracellular HCV RNA isolated from culture supernatants (F) were analyzed by qRT-PCR. (E) Total cell lysates harvested at 48 h after Jc1 infection were immunoblotted with the indicated antibodies. (G) Intracellular infectious HCV particles were prepared by 4 rounds of freeze and thaw treatments from cells treated as in figure legend to C. Neg indicates cells infected with intracellular HCV isolated from the Negative siRNA-transfected cells. PC denotes cells infected with intracellular HCV isolated from the PC siRNA-transfected cells. Pos denotes cells infected with intracellular HCV isolated from the Positive siRNA-transfected cells. (H) Extracellular infectious HCV particles were prepared from the culture media in cells treated as in figure legend to C. Neg indicates cells infected with extracellular HCV isolated from the Negative siRNA-transfected cells. PC denotes cells infected with extracellular HCV isolated from the PC siRNA-transfected cells. Pos denotes cells infected with extracellular HCV isolated from the Positive siRNA-transfected cells. (G, H) Naïve Huh7.5 cells were then infected with intracellular infectious HCV (G) and extracellular infectious HCV (H). Total cell lysates harvested at 2 days postinfection were immunoblotted to determine the indicated protein levels, and HCV RNA levels were analyzed by qRT-PCR (top panels). Naïve Huh7.5 cells treated as described above were analyzed for immunofluorescence using anti-NS5A antibody (bottom panels). Cells were counterstained with DAPI to label nuclei. Samples were analyzed for immunofluorescence staining using a Zeiss LSM 700 laser confocal microscopy system. Asterisks indicate significant differences (*, *P*<0.05, **, *P*<0.01) from the value for the negative control. Error bars indicate standard deviations.

To investigate the role of PC in HCV propagation, HCV subgenomic replicon cells were transfected with the siRNA pools, including negative, PC, and positive, respectively. Silencing of PDI, one of binding proteins with NS5A (Fig. 1), had no effect on intracellular HCV RNA level (Fig. S1). Likewise, silencing of PC showed little effect on intracellular HCV RNA level (Fig. 3A). Fig. 3B showed that HCV protein expression levels were efficiently suppressed by positive siRNA without affecting cellular protein levels of actin and PC. When PC was silenced by siRNA pool containing four siRNA constructs targeting different sites of PC, HCV protein expression levels were not pronouncedly decreased as compared with the cells treated with the negative control siRNAs (Fig. 3B, lane 1 versus lane 2). This result suggests that PC is not involved in the replication stage of the HCV life cycle. To further verify this finding, we investigated the role of PC in HCV propagation using HCVcc-infected cells. Huh7.5 cells transfected with the indicated siRNAs for 2 days were infected with Jc1. Fig. 3C showed that treatments of siRNAs induced no cell toxicity as measured by an MTT assay. Knockdown of PC had no effect on both intracellular HCV RNA (Fig. 3D) and protein levels (Fig. 3E). However, extracellular HCV RNA level (Fig. 3F) was significantly decreased at 48 h postinfection as compared to negative siRNA control. To further verify these results, we isolated both intracellular HCV and extracellular HCV from supernatant of primary infected cells. Naïve Huh7.5 cells were then infected with either intracellular HCV or extracellular HCV and the effect of PC knockdown on HCV propagation was analyzed in secondary infected cells. We demonstrated that silencing of PC pronouncedly impaired HCV RNA and protein levels in intracellular HCV-infected cells (Fig. 3G) similar to extracellular HCV-infected cells (Fig. 3H). This was further confirmed by immunofluorescence assays. Since HCV RNA and proteins levels were significantly reduced in PC knockdown cells as compared to negative siRNA-treated cells in secondary infection experiments, these data implied that PC might be involved in the stage of virion packaging and release in the HCV life cycle.

### NS5A Modulates PC Expression

**Figure 4:**
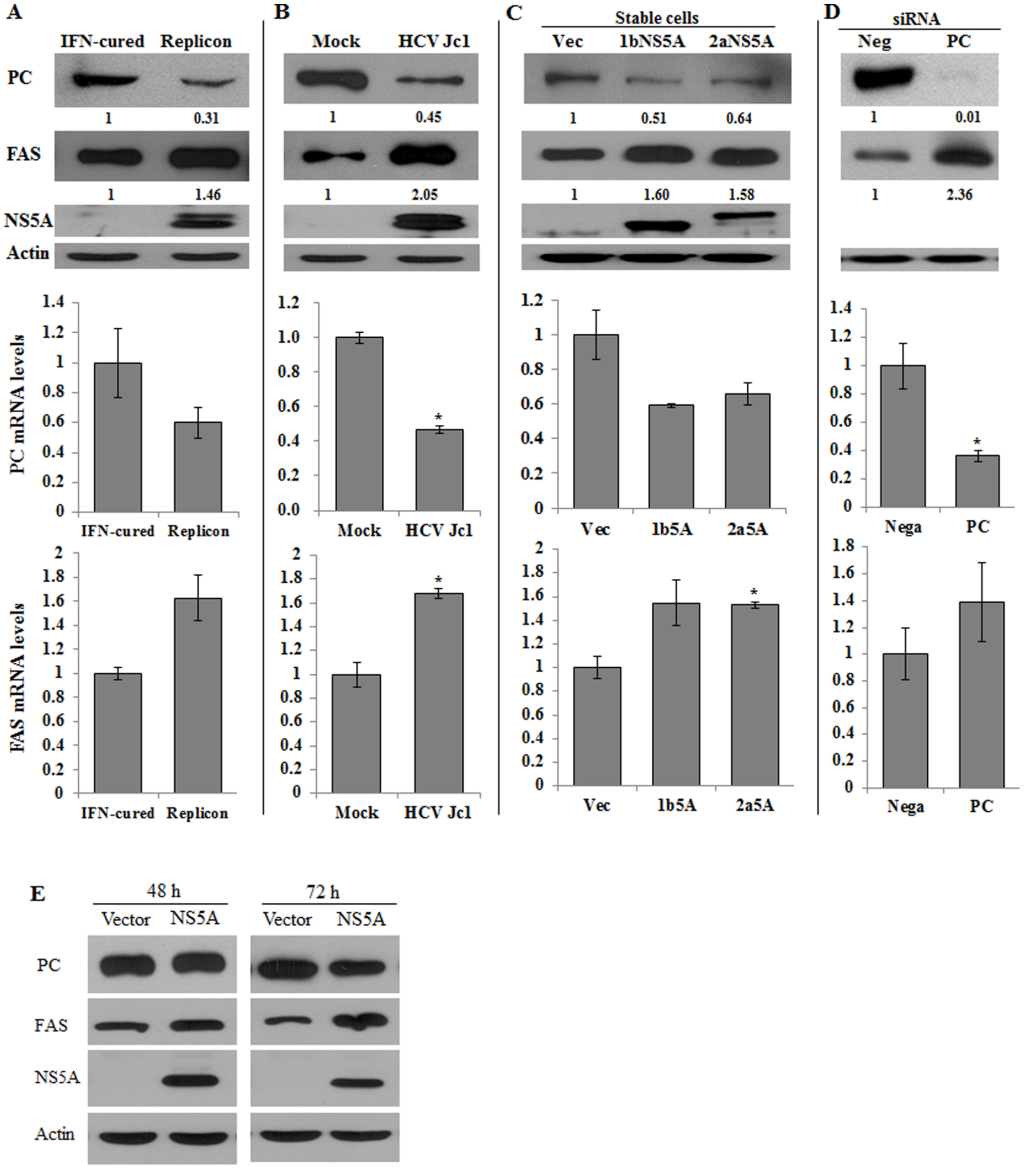
NS5A regulates PC expression level. (A–C) PC expression level is decreased, whereas FAS expression level is increased in cells expressing NS5A protein. (A) Total cell lysates harvested from either IFN-cured cells or replicon cells were immunoblotted with the indicated antibodies (top panel). Total RNAs isolated from either IFN-cured cells or replicon cells were quantitated for PC mRNA levels (middle panel) and FAS mRNA levels (bottom panel) by qRT-PCR. (B) Huh7.5 cells were either mock infected or infected with Jc1. Total cell lysates harvested at 5 days postinfection were immunoblotted with the indicated antibodies (top panel). Total RNAs isolated from either mock infected or Jc1 infected cells were quantitated for PC mRNA levels (middle panel) and FAS mRNA levels (bottom panel) by qRT-PCR using data from three independent experiments. Asterisks indicate significant differences (*, *P*<0.05) from the value for the control. Error bars indicate standard deviations. (C) Equal amounts of cell lysates harvested from either vector stable or NS5A stable cells derived from genotype 1b or 2a were immunoblotted with the indicated antibodies (top panel). Total RNAs isolated from the indicated stable cells were quantitated for PC mRNA levels (middle panel) and FAS mRNA levels (bottom panel). Asterisks indicate significant differences (*, *P*<0.05) from the value for the control. (D) Huh7.5 cells were transfected with siRNAs of either negative or PC. At 3 days after transfection, cells were harvested and analyzed by immunoblotting with the indicated antibodies (top panel). Actin was used as a loading control. Total RNAs isolated from the indicated cells were quantitated for PC mRNA levels (middle panel) and FAS mRNA levels (bottom panel). Asterisks indicate significant differences (*, *P*<0.05) from the value for the control. (E) Huh7.5 cells were transiently transfected with either pEF6 empty vector or pEF6-NS5A-Myc expression plasmid. Cell lysates harvested at 48 h or 72 h after transfection were immunoblotted with the indicated antibodies.

Because functionally clustered gene expressions are serially changed in HCV patients [20] and both PC and fatty acid synthase (FAS) are lipogenic enzymes, we compared both PC and FAS expression levels in both HCV replicon cells and HCV-infected cells. We showed that both protein and mRNA levels of PC were decreased in replicon cells and in HCV-infected cells (Fig. 4A and 4B). Since NS5A interacted with PC, we speculated that NS5A might regulate PC expression level. To verify this, we determined both protein and mRNA level of PC in Huh7 cells stable expressing NS5A derived from either genotype 1b or 2a. Indeed, both NS5A stable cells expressed lower levels of PC protein and mRNA (Fig. 4C). However, FAS expressions were increased in NS5A expressing cells, including HCV replicon cells and HCV-infected cells. Interestingly, FAS expression was pronouncedly increased in PC silenced cells (Fig. 4D). It was noteworthy that PC expression levels were unchanged until 2 days postinfection and then were gradually reduced (Fig. S2). In fact, PC expression levels were dramatically decreased at day 5 postinfection in Jc1-infected cells (Fig. 4B). We further demonstrated that PC protein expression level was gradually decreased, whereas FAS level was gradually increased in Huh7.5 cells transiently expressing NS5A (Fig. 4E). Collectively, these data indicate that NS5A, either alone or in the context of the full-length HCV polyprotein, modulates FAS and PC expression levels.

**Figure 5:**
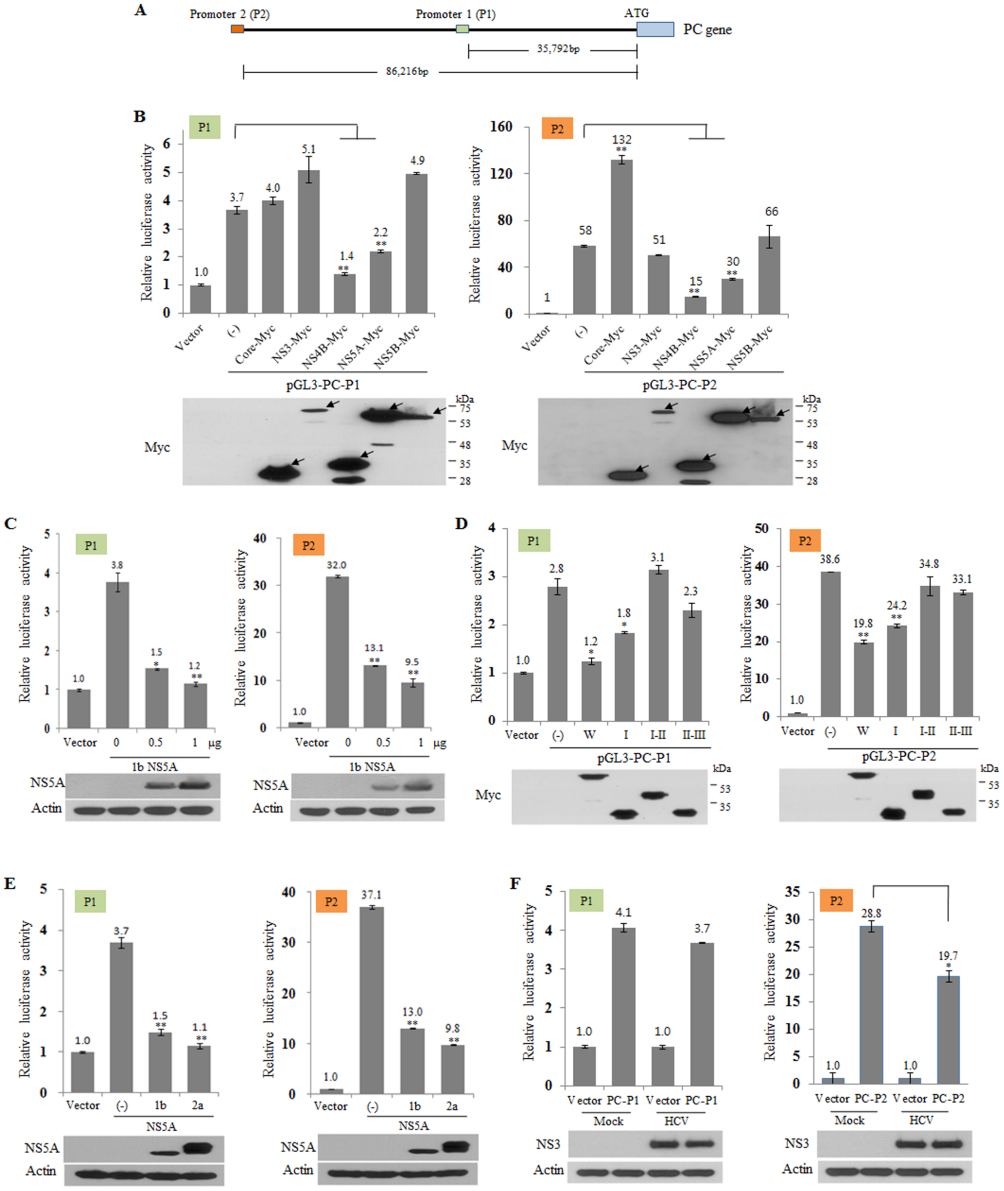
HCV proteins modulate transcriptional activity of PC. (A) Schematic diagram of two PC promoters, P1 and P2, used in this study. (B) HEK293T cells were transfected with either P1-Luc (left panel) or P2-Luc (right panel) reporter plasmid together with the indicated HCV protein expression plasmid. At 24 h after transfection, cells were harvested and luciferase activities were determined (top panels). Protein expressions were determined by anti-Myc antibody as shown in arrows (bottom panels). Vector indicates pGL3 empty vector. (-) denotes pGL3-PC in the absence of HCV protein (C) HEK293T cells were transfected with either P1-Luc (left panel) or P2-Luc (right panel) reporter plasmid together with increasing amounts of NS5A expression plasmid (genotype 1b). Cells were harvested at 24 h after transfection and then luciferase activities were determined (top panels). NS5A expressions were determined by anti-NS5A antibody (bottom panels). (D) HEK293T cells were transfected with either P1-Luc (left panel) or P2-Luc (right panel) reporter plasmid together with Myc-tagged mutant constructs of NS5A. At 24 h after transfection, cells were harvested and luciferase activities were determined. (E) HEK293T cells were transfected with either P1-Luc (left panel) or P2-Luc (right panel) reporter plasmid together with NS5A plasmid derived from either genotype 1b or 2a. At 24 h after transfection, cells were harvested and luciferase activities were determined. (F) Huh7.5 cells were either mock infected or infected with HCV Jc1 for 4 h. At 5 days postinfection, cells were transfected with either vector or P1-Luc (left panel), or vector or P2-Luc (right panel) reporter plasmid. Cells were further cultured for 2 days and then luciferase activities were determined. Protein expressions were verified by immunoblot analysis using anti-NS3 and anti-actin antibody, respectively. Luciferase activities were normalized based on β-galactosidase activities. Asterisks indicate significant differences (*, *P*<0.05, **, *P*<0.01) from the activity for the control.

PC gene maps on human chromosome 11 at 11q13.4-q13.5 on the reverse strand [10], [11]. The PC gene derived from either mouse or rat has been well characterized for its transcriptional regulation. Two alternative promoters, the proximal and the distal, of human PC have also been identified in liver [10]. Both P1 (pGL3-PC-P1) and P2 (pGL3-PC-P2) promoters of human PC (Fig. 5A) were constructed as described in Materials and Methods. HEK293T cells were transfected with either pGL3-PC-P1 or pGL3-PC-P2, and then promoter activities were determined. We showed that P1 promoter activity was increased ∼4-fold and P2 activity was increased ∼65-fold as compared with pGL3 empty vector (Fig. S3). To verify which HCV protein was involved in PC regulation, HEK293T cells were cotransfected with each of the indicated Myc-tagged HCV protein expression plasmid together with either P1 or P2 promoter construct. Fig. 5B showed that promoter activities of PC were decreased by both NS4B and NS5A proteins. Interestingly, promoter activity of P2 was significantly increased by core (Fig. 5B, right panel). Since PC expression level was decreased in cells expressing NS5A, we further investigated the effect of NS5A on PC down-regulation. As shown in Fig. 5C, promoter activities of both P1 and P2 were significantly decreased by NS5A in a dose dependent manner. To investigate which domain of NS5A was involved in PC regulation, we analyzed PC promoter activities using various NS5A mutants. Fig. 5D showed that both P1 and P2 promoter activities were significantly suppressed by both wild-type and I mutant bearing binding region for PC, indicating that protein interplay between NS5A and PC may be involved in the regulation of PC promoter activities. We further showed that promoter activities of both P1 and P2 were significantly reduced by NS5A derived from genotype 1b and 2a (Fig. 5E). Finally, we investigated promoter activities of both P1 and P2 in the context of the full-length HCV polyprotein in HCV-infected cells. Huh7.5 cells were either mock infected or infected with Jc1 for 5 days and then promoter activity was determined. As demonstrated in Fig. 5F, P1 reporter activity was unchanged in Jc1-infected cells as compared to the mock-infected cells (left panel). However, transcriptional activity was significantly decreased in cells transfected with P2 promoter in Jc1-infected cells (Fig. 5F, right panel). These data suggest that HCV modulates P2 promoter activity via NS5A protein for its own propagation.

Since FAS level was increased, while PC level was decreased in HCV infected cells, we further investigated whether triglyceride level was also altered by HCV infection. Using triglyceride assay kit (Bioassay Systems, Haywards, CA), we demonstrated that triglycerides were accumulated in HCV infected cells as compared with mock infected cells (Fig. S4). We further showed that triglyceride level was higher in NS5A stable cells as compared with vector stable cells (Fig. S5). These data suggest that NS5A may modulate PC and triglyceride levels to facilitate virion assembly in HCV-infected cells.

## Discussion

Exploitation of host cellular machineries is a common strategy shared by all viruses. HCV recruits replication complexes to lipid droplets for producing infectious viruses [10]. NS5A forms part of the HCV RNA replication complex. NS5A has been shown to interact with various cellular proteins to modulate cell growth and cellular signaling pathways. To identify cellular factors necessary for HCV propagation, we employed a tandem affinity purification assay. By LC-MS/MS of the coprecipitated protein, PC was identified as one of the binding partners for NS5A protein. PC interacted with NS5A derived from both genotype 1b and 2a. NS5A interacted with PC through the biotin carboxylase domain of PC and the domain I of NS5A. We further demonstrated protein interplay between PC and NS5A in the context of HCV replicating cells.

PC is a genomic encoded mitochondrial enzyme [5]. Since mitochondria are multifunctional organelles involved in energy production, apoptosis, immune response, and aging, they often become a target of viruses [21]. Protein interplay between virus and mitochondria may cause pathogenic effect on host cells. In fact, mitochondrial localization of viral proteins results in disruption of membrane potential. For example, hepatitis B virus X protein (HBx) localizes on mitochondria and regulates mitochondrial membrane potential in hepatocytes [22]. Viral protein R (Vpr) of HIV-1 induced mitochondrial membrane permeabilization and apoptosis [23]. Cytomegalovirus cell death suppressor vMIA prevented Bax-mediated mitochondrial membrane permeabilization and thus inhibited apoptosis [24]. HCV NS4A also induced collapse of mitochondrial membrane potential and release of cytochrome c into the cytoplasm, which then induced apoptosis [25]. In addition, HCV NS5A was colocalized with FKBP38 in mitochondria and inhibited apoptosis [26]. Recently, Romero-Brey et al. reported that the amount of NS5A localized in the mitochondria was equivalent to that in the ER [27]. The authors demonstrated that signal intensity determined by Pearson’s correlation coefficient of ER and NS5A was similar to that of mitochondria and NS5A. In the present study, we verified that both PC and NS5A were colocalized in the mitochondria as determined by immunofluorescence assay, and immunoblot analysis using mitochondrial fraction. Collectively, these data indicated that a fraction of NS5A was colocalized in the mitochondria where it interacted with PC.

PC plays an important role in modulating fractional distribution of intracellular acetyl-CoA between the TCA cycle and fatty acid synthesis [5], [6]. Since PC deficiency leads to lack of oxaloacetate, the liver of some PC deficient patients is enlarged and lipid droplets are accumulated in hepatocytes [28], [29]. Decreased oxaloacetate availability leads to failure of hepatic acetyl-CoA oxidation, which is then diverted into ketone body formation such as acetoacetate in mitochondria and it will be exported to the cytosol. Acetoacetate in the cytosol is converted again to acetyl-CoA, which is used as a source for fatty acid synthesis. Interestingly, PC expression is downregulated in fibrotic tissues of liver in HCV infected patients [20]. We also showed that the transcriptional activity of PC was downregulated in cells expressing NS5A, in HCV replicon cells, and in HCV infected cells, whereas FAS level was elevated in HCV infected cells, which resulted in triglyceride accumulation as reported previously [30]. These data suggest that HCV may regulate PC expression to increase acetyl-CoA, a building block of fatty acid, in the cytosol to accumulate lipid droplets for favor its own propagation.

The PC gene of rat is regulated by two alternative promoters, the proximal and the distal [5]. The distal promoter is active in most tissues, whereas the proximal promoter is active in gluconeogenic and lipogenic tissues, indicating that these two promoters work under different physiological circumstances. Transcription of human PC has been identified 11 different mRNAs, 9 alternatively spliced variants and 2 unspliced forms, which is regulated by two promoters. In the present study, two promoters of human PC were cloned to investigate the transcriptional activity in the presence of HCV proteins. Two promoters were differentially modulated by HCV proteins. Transcriptional activities of both P1 and P2 promoters were down-regulated by NS4B and NS5A. Interestingly, P2 promoter (distal promoter) activity was increased by core. In HCVcc-infected cells, the transcriptional activity of P2 but not P1 was down-regulated. These data suggest that P2 promoter plays an important role in the regulation of PC transcription during HCV infection. It has been previously reported that P2 promoter was regulated by hepatocyte nuclear factor 3b/Foxa2 and upstream stimulatory factors in the rat [31]. However, the regulatory mechanism of human PC expression has not been fully characterized yet.

Although NS5A down-regulated PC expression level, silencing of PC reduced infectious particle production in HCV infected cells. This may explain that basal level of PC is also required for HCV propagation. Because FAS level was upregulated in PC-silenced cells, we temped to speculate that HCV might modulate cellular homeostasis temporally and spatially by balancing glycolysis and lipogenesis to facilitate viral propagation. In the replication stage of the HCV life cycle, PC may be activated to generate more oxaloacetates to promote HCV replication, whereas NS5A may suppress PC activity in the assembly stage of the HCV life cycle to stimulate lipogenesis. These data suggest that NS5A may play a role as an on/off switch to control HCV propagation by interacting with PC protein.

## Supporting Information

Figure S1 **Knockdown of PDI had no effect on HCV RNA and protein levels in HCV replicon cells.** (A) Huh7 cells harboring HCV replicon were transfected with 10 nM of the indicated siRNA duplexes for 3 days. Total RNAs were extracted and intracellular HCV RNA was analyzed by qRT-PCR. (B) Total replicon cell lysates harvested at 3 days after siRNA transfection were immunoblotted with the indicated antibodies.
(TIF)

Figure S2 **Huh7.5 cells were either mock infected or infected with Jc1.** Total cell lysates harvested at the indicated time points were immunoblotted with anti-PC, anti-NS5A, anti-NS3, and anti-actin antibodies, respectively.
(TIF)

Figure S3 **(A) HEK293T cells were transfected with either pGL3-basic or pGL3-P1 reporter plasmid. At 24 h after transfection, cells were harvested and luciferase activities were determined.** (B) HEK293T cells were transfected with either pGL3-basic or pGL3-P2 reporter plasmid. At 24 h after transfection, luciferase activities were determined. EV, empty vector.
(TIF)

Figure S4 **The lipid is accumulated in HCV infected cells.** Huh7.5 cells were either mock infected or infected with Jc1. At day 5 postinfection, triglyceride levels were analyzed according to the manufacturer’s instructions.
(TIF)

Figure S5 **The lipid is accumulated in NS5A stable cells.** Huh7 cells were transfected with pEF6-empty vector, pEF6-1b NS5A, or pEF6-2a NS5A and were cultured for 4 weeks in the presence of 5 µg/ml blasticidin. Single positive clones were selected by immunoblot analysis. Triglyceride levels were analyzed according to the manufacturer’s instructions.
(TIF)
